# Mini Mental State Examination and Logical Memory scores for entry into Alzheimer’s disease trials

**DOI:** 10.1186/s13195-016-0176-z

**Published:** 2016-02-22

**Authors:** Kimberly R. Chapman, Hanaan Bing-Canar, Michael L. Alosco, Eric G. Steinberg, Brett Martin, Christine Chaisson, Neil Kowall, Yorghos Tripodis, Robert A. Stern

**Affiliations:** Boston University Alzheimer’s Disease and CTE Center, 72 East Concord Street, Suite B7800, Boston, MA 02118 USA; Department of Neurology, Boston University School of Medicine, Boston, MA 02118 USA; Data Coordinating Center, Boston University School of Public Health, Boston, MA 02118 USA; Department of Biostatistics, Boston University School of Public Health, Boston, MA 02118 USA; Department of Pathology, Boston University School of Medicine, Boston, MA 02118 USA; Neurology Service, VA Boston Healthcare System, Boston, MA 02130 USA; Department of Neurosurgery, Boston University School of Medicine, Boston, MA 02118 USA; Department of Anatomy & Neurobiology, Boston University School of Medicine, Boston, MA 02118 USA

**Keywords:** Alzheimer’s disease, Clinical trials, MMSE, Logical Memory, Eligibility, Mild cognitive impairment, Neurodegenerative disease

## Abstract

**Background:**

Specific cutoff scores on the Mini Mental State Examination (MMSE) and the Logical Memory (LM) test are used to determine inclusion in Alzheimer’s disease (AD) clinical trials and diagnostic studies. These screening measures have known psychometric limitations, but no study has examined the diagnostic accuracy of the cutoff scores used to determine entry into AD clinical trials and diagnostic studies.

**Methods:**

ClinicalTrials.gov entries were reviewed for phases II and III active and recruiting AD studies using the MMSE and LM for inclusion. The diagnostic accuracy of MMSE and LM-II cutoffs used in AD trials and diagnostic studies was examined using 23,438 subjects with normal cognition, mild cognitive impairment (MCI), and AD dementia derived from the National Alzheimer’s Coordinating Center database.

**Results:**

MMSE and LM cutoffs used in current AD clinical trials and diagnostic studies had limited diagnostic accuracy, particularly for distinguishing between normal cognition and MCI, and MCI from AD dementia. The MMSE poorly discriminated dementia stage.

**Conclusions:**

The MMSE and LM may result in inappropriate subject enrollment in large-scale, multicenter studies designed to develop therapeutics and diagnostic methods for AD.

## Background

Alzheimer’s disease (AD) clinical trials and diagnostic studies are responsible for the testing and development of therapeutics and diagnostic methods for AD. These large-scale, multicenter studies must have strict inclusion criteria to accurately identify and discriminate normal cognition, mild cognitive impairment (MCI), and AD dementia and recruit the population of interest to facilitate internal and external validity. This, however, is no straightforward task. Although there have been great gains in the development of biomarkers for the accurate in vivo diagnosis and early detection of AD (e.g., lumbar puncture, positron emission tomography) [1–3], these are invasive procedures typically conducted following initial screening methods. Instead, investigators in AD clinical trials and diagnostic studies often initially rely on brief cognitive screening tests to detect cognitive impairment and classify patients, using a variety of research-derived cut scores, as having normal cognition, MCI, or dementia. The Mini Mental State Examination (MMSE) [4] and the Wechsler Memory Scale (WMS) Logical Memory (LM) test [5] are two screening measures commonly used to determine inclusion in these studies.

The use of the MMSE and LM in AD clinical trials and diagnostic studies to ascertain diagnostic status and determine inclusion may be methodologically problematic. Numerous studies have demonstrated the psychometric limitations of the MMSE, such as large ceiling and floor effects, and sensitivity to practice effects [6–8]. The utility of the MMSE in detecting MCI and AD dementia is indeed limited [9–11]. Perneczky et al. [12] examined the correspondence between the MMSE and Clinical Dementia Rating (CDR) scores and found the MMSE lacked accuracy in the identification of patients with MCI or mild AD dementia.

Scores on the delayed recall dimension of LM (LM-II) can also lack diagnostic utility when administered in isolation. LM-II is associated with significant learning biases [13], and practice effects may undermine its detection of impairment, particularly among potential AD trial subjects who have had repeated exposure to LM. For example, LM has been administered annually to all participants in the National Institutes of Health (NIH)-funded Alzheimer’s Disease Centers (ADCs), as part of the National Alzheimer’s Coordinating Center (NACC) Uniform Data Set (UDS), for approximately 20 years [14]. The consortium of these centers is an important source of enrollment for AD clinical trials. The ability of the LM relative to other tests to accurately detect AD has also been questioned [15], and healthy older adults frequently demonstrate impairments on LM retention [16]. Performance on LM may also be relatively more sensitive to executive dysfunction than episodic memory [17].

Given the diagnostic and psychometric limitations of the MMSE and LM, many AD clinical trials and diagnostic studies in which these instruments are used to determine eligibility may be inappropriately including or excluding subjects. This could influence the reliability and validity of study outcomes due to sampling biases. The recent phase III bapineuzumab and solanezumab trials both included the MMSE as part of study entry criteria, and both failed to meet primary efficacy endpoints [18, 19]. (In fact, no new compounds for the treatment of AD have been approved by the U.S. Food and Drug Administration since 2003.) Although research from the Alzheimer’s Disease Neuroimaging Initiative (ADNI) has been at the forefront of the development of diagnostic methods and biomarkers for AD, ADNI also relies on MMSE and LM scores to determine eligibility.

The extent to which MMSE and LM scores are being used as eligibility criteria in AD clinical trials and diagnostic studies is unclear, and no study has examined whether the cutoffs used in these studies accurately correspond to AD spectrum clinical diagnoses. The purpose of the present study was twofold: (1) to identify all active and recruiting phases II and III AD clinical trials and diagnostic studies in the United States to determine the extent to which the MMSE and LM are used as eligibility criteria and to identify the cutoff scores used to ascertain AD diagnostic category; and (2) to exploit the large NACC database to determine the correspondence between MMSE and LM cutoff scores used in current clinical trials and diagnostic studies and AD spectrum diagnoses made by multidisciplinary diagnostic conference teams. The MMSE is often used to determine dementia severity in clinical and research settings, and past work [12] suggests this may be problematic due to the weak correspondence between the MMSE and the CDR (the gold standard for rating dementia severity), particularly at the mild end of the disease spectrum. Therefore, in the present study we sought, as a secondary aim, to replicate and expand upon the previous smaller-scale study on the MMSE and CDR [12] by examining their correspondence in the large NACC dataset.

## Methods

### Search criteria

We first examined the extent to which the MMSE and LM are used in AD clinical trials and diagnostic studies, as well as the cutoff scores employed in these studies. To do so, all phases II and III recruiting and active AD trials were identified in the ClinicalTrials.gov database. The search was limited to U.S. trials that listed “Alzheimer’s disease” as a keyword. Inclusion criteria as they pertained to MMSE and LM and their cutoffs were obtained from the inclusion description under “Eligibility.”

### Subjects

The diagnostic accuracy of MMSE and LM cutoffs used in AD trial and diagnostic studies was tested using subjects in the NACC database diagnosed with normal cognition (*n* = 10,741), MCI (*n* = 5883), and AD dementia (*n* = 6814). The NACC, established by the National Institute on Aging in 1999 to promote collaborative AD research, is a publicly accessible, longitudinal database of standardized clinical data gathered from 34 past and present ADCs across the United States. The regional ADCs are based in university medical centers, and recruitment is carried out via neurology referrals and community outreach. Each year beginning in 2005, the ADCs have contributed standardized cognitive, behavioral, and functional data for each participant to a UDS that now forms the NACC-UDS database. For full descriptions of the NACC-UDS, please refer to publications by Weintraub et al. [14], Beekly et al. [20, 21], and Morris et al. [22]. Before engagement in the research registry, written informed consent was obtained by all study participants or their legally authorized representatives. All aspects of the study adhered to necessary ethical guidelines and were approved by the local ADC’s human subjects review board.

A formal data request to NACC for this study was approved (proposal ID 606), and data were provided on 28 September 2015. The sample was restricted to initial visits of subjects between the ages of 50 and 100 years with a diagnosis of normal cognition, MCI, or primary possible or probable AD dementia. Baseline evaluations for the current sample occurred between 2005 and 2015. Data queried included the UDS cognitive test battery (see below for version), diagnostic status, CDR score, and demographic variables. The sample was further restricted to those who completed the English version of the MMSE. See Table 1 for study variables.

**Table 1:** NACC sample characteristics

	Normal cognition	MCI	AD dementia	*P* value
Number of subjects	10,741	5883	6814	–
Age, yr, mean (SD)	71.92 (9.40)	73.87 (8.93)	75.28 (9.28)	<0.001
Education,^a^ yr, mean (SD)	15.74 (2.86)	15.21 (3.21)	14.53 (3.30)	<0.001
Female sex, *n* (%)	7047 (65.6)	2907 (49.4)	3689 (54.1)	<0.001
White race,^a^ *n* (%)	8713 (81.3)	4682 (79.8)	5639 (82.8)	<0.01
Global CDR, mean (SD)	0.05 (0.15)	0.46 (0.17)	1.02 (0.57)	<0.001
MMSE, mean (SD)	28.88 (1.41)	27.11 (2.43)	20.92 (5.50)	<0.001
Logical Memory-II raw score, mean (SD)	12.17 (4.13)	6.92 (4.64)	1.82 (2.88)	<0.001

*Abbreviations*: *NACC* National Alzheimer’s Coordinating Center, *CDR* Clinical Dementia Rating, *MMSE* Mini Mental State Examination, *MCI* mild cognitive impairment, *AD* Alzheimer’s disease, *SD* standard deviation

Post hoc analyses showed significant differences across all three groups

^a^Due to missing data, sample size for education is reduced to 10,695 for normal cognition, 5863 MCI, and 6779 for AD dementia and sample size for race is 10,721 normal cognition, 5869 MCI, and 6807 AD

### Diagnostic categories

For the current NACC sample, 23.0 % of neurological diagnoses were made by a single clinician and 77.0 % of the diagnoses were assigned through multidisciplinary diagnostic consensus conferences composed of neurologists, neuropsychologists, geriatricians, and geriatric psychiatrists. Consensus diagnoses were made following presentation and discussion of all examinations, UDS (and other) test findings (including neuroimaging and other biomarkers, if available), and psychosocial and medical history. At the time of data collection for this study, AD dementia was diagnosed on the basis of the National Institute of Neurological and Communicative Disorders and Stroke/Alzheimer’s Disease and Related Disorders Association criteria [23]. MCI diagnosis was based on criteria defined by Winblad et al. [24].

### Measures

#### Mini Mental State Examination

The MMSE is a 30-item assessment of global cognitive status that taps into domains such as orientation, concentration, attention, verbal learning (without delayed recall), naming, and visuoconstruction [4]. Despite its weaknesses, the MMSE has long been used to detect and monitor dementia progression.

#### Logical Memory test

The LM subtest of the WMS-R is a standardized assessment of narrative episodic memory [5]. A short story is orally presented, and the examinee is asked to recall the story verbatim (immediate recall). Approximately 20 or 30 min later, free recall of the story is again elicited (delayed recall). Of the NACC sample, 11,569 subjects were administered the UDS cognitive battery version 1 and 11,869 were given version 2. Between 2005 and 2007, two version 1 examinations were administered (1.1 and 1.2), with version 1.1 having a delayed story recall of 30 min and version 1.2 being 20 min. The UDS version 2 retained the 20-min recall. For all UDS versions, LM Story A delayed recall was used and the only difference from version 1.1 to versions 1.2 and 2 was the delay interval. Of note, we were unable to distinguish between subjects who received versions 1.1 and 1.2, but the differences in the delay intervals have been shown not to be associated with number of units recalled [14].

#### Clinical Dementia Rating

The CDR is a widely used, valid, and reliable tool for staging dementia severity [25–27]. Specifically, the CDR is standardized for multicenter use, has demonstrated good interrater reliability and criterion validity, and has been shown to predict neuropathology [26]. In fact, even without an informant, recent work in community-dwelling elderly shows the CDR exhibits strong internal consistency (Cronbach’s α 0.83–0.84) and good interrater reliability (0.95 for global rating) and test-retest reliability (κ = 0.80 for global rating) [28]. The CDR assesses the extent of a person’s impairment in six domains: memory, orientation, judgment/problem-solving, community affairs, home and hobbies, and personal care. An algorithm is used to create an overall rating of impairment severity: 0 (no dementia), 0.5 (questionable dementia), 1.0 (mild dementia), 2.0 (moderate dementia), or 3.0 (severe dementia). Typically, a score of 0.5 is given to individuals with a diagnosis of MCI [25].

### Statistical analyses

Subjects were excluded for missing data on the MMSE or LM that was due to physical, cognitive, or behavioral (including refusal) problems that interfered with testing. Receiver operating characteristic (ROC) curves were examined to evaluate the accuracy [area under the ROC curve (AUC)] of the MMSE and LM cutoffs used in AD clinical trials and diagnostic studies (results presented below) in distinguishing the diagnostic groups. The MMSE and LM-II were transformed to binary variables (i.e., above and below the identified cutoff) and served as the test variable. An AUC value of 0.75 was considered to be clinically meaningful [29]. ROC curve analyses were repeated with MMSE and LM as continuous test variables to obtain the sensitivity and specificity values of the cutoffs used in AD clinical trials and diagnostic studies. Positive and negative predictive values (PPVs and NPVs, respectively) were then calculated to determine the diagnostic accuracy of the cutoffs in the NACC sample. The prevalence of MCI and AD dementia used for the calculation of PPV and NPV was based on the prevalence of MCI and AD dementia in this sample and, in some instances, varied according to the age or educational group to which analyses were restricted.

κ Statistics were used to examine the level of agreement between the MMSE and CDR groups. MMSE cutoffs of 30, 29–26, 25–21, 20–11, and 10–0 have previously been shown to map onto CDR scores (0, 0.5, 1.0, 2.0, and 3.0, respectively) and thus were used in this study to define no, questionable, mild, moderate, and severe dementia, respectively [12]. Standard convention was used in the interpretation of κ values in terms of level of agreement [30].

## Results

### Prevalence of MMSE and LM as inclusion criteria in AD clinical trials and diagnostic studies

There were 111 phases II and III AD trials and diagnostic studies that were listed as recruiting or active. Of those 111, 64 (57.7 %) used the MMSE for eligibility criteria, including randomized controlled treatment trials “Effect of Passive Immunization on the Progression of Mild Alzheimer’s Disease: Solanezumab (LY2062430) Versus Placebo” (sponsored by Eli Lilly and Company) and “Anti-Amyloid Treatment in Asymptomatic Alzheimer’s Disease (A4 Study)” (sponsored by Eli Lilly and Company, Alzheimer’s Disease Cooperative Study collaborator). The major multisite diagnostic study, Alzheimer’s Disease Neuroimaging Initiative 2 (ADNI 2) (funded by the National Institute on Aging, the National Institute of Biomedical Imaging and Bioengineering, and private sector contributions facilitated by the Foundation of the National Institutes of Health), was also found to use the MMSE. MMSE cutoffs ranging from 3–14 to >27, but ≤24 and/or ≤26 most commonly defined the clinical spectrum of AD. A majority of studies that use 26 primarily target subjects with MCI or mild AD dementia.

Seven recruiting and active AD clinical trials use LM to determine eligibility, and five of the seven use both the MMSE and LM. All trials use the delayed recall score of LM (i.e., LM-II). Notable trials include the “Anti-Amyloid Treatment in Asymptomatic Alzheimer’s Disease (A4 Study)” and “A Placebo-controlled, Double-blind, Parallel-group, Bayesian Adaptive Randomization Design and Dose-Regimen-find Study to Evaluate Safety, Tolerability and Efficacy of BAN2401 in Subjects with Early Alzheimer’s Disease” (sponsored by Eisai Inc.). There was inconsistency with the WMS version used: the BAN2401 uses WMS-IV, but other trials use the WMS-R or the WMS-III. ADNI 2 also uses LM-II (WMS-R) to determine study eligibility. ADNI 2 and the A4 and BAN2401 trials use both the LM-II and MMSE.

Regarding LM-II cutoffs, the A4 trial uses 6–18 to define asymptomatic AD. The BAN2401 trial cutoffs are age-adjusted and include 50–64 years: ≤15; 65–69 years: ≤12; 70–74 years: ≤11; 75–79 years: ≤9; and 80–90 years ≤7. ADNI 2 implements the following LM-II education-based cutoffs:

- 16 years of education: normal ≥9; early MCI = 9–11; AD ≤8
- 8–15 years of education: normal ≥5; early MCI = 5–9; AD ≤4
- 0–7 years of education: normal ≥3; early MCI = 3–6; AD ≤2

### AUC, PPV, and NPV for MMSE cutoff scores

#### AUC

On the basis of the above-described search results, we tested the accuracy of MMSE scores ≤24 and/or ≤26 in distinguishing normal cognition from AD dementia and MCI, respectively, and then MCI from AD dementia. Table 2 presents AUC values. The accuracy of an MMSE ≤26 was suboptimal for MCI, but an MMSE of ≤24 was adequate for detecting AD dementia.

**Table 2:** AUC values for MMSE and LM-II cutoffs used in AD clinical trials and diagnostic studies in NACC subjects

	Normal cognition vs. MCI	Normal cognition vs. AD dementia	MCI vs. AD dementia
MMSE scores			
MCI cutoff score ≤26	0.63	–	–
AD dementia cutoff score ≤24	–	0.85	0.80
LM-II score (A4 Trial)			
Cutoff score <6	0.67	0.92	0.74
LM-II score (BAN2401)			
50–64 years old			
Cutoff score ≤15	0.61	0.64	0.53
65–69 years old			
Cutoff score ≤12	0.68	0.76	0.58
70–74 years old			
Cutoff score ≤11	0.71	0.78	0.57
75–79 years old			
Cutoff score ≤9	0.72	0.84	0.62
80–90 years old			
Cutoff score ≤7	0.72	0.88	0.67
LM-II score (for early MCI in ADNI 2)			
16+ years of education			
Cutoff score ≤11	0.72	–	–
8–15 years of education			–
Cutoff score ≤9	0.70	–	–
0–7 years of education			–
Cutoff score ≤6	0.69	–	–
LM-II score (for AD dementia in ADNI 2)			
16+ years of education			
Cutoff score ≤8	–	0.90	0.67
8–15 years of education			
Cutoff score ≤4	–	0.92	0.76
0–7 years of education			
Cutoff score ≤2	–	0.87	0.76

*Abbreviations*: *NACC* National Alzheimer’s Coordinating Center, *MMSE* Mini Mental State Examination, *LM-II* Wechsler Memory Scale Logical Memory-II, *MCI* mild cognitive impairment, *ADNI 2* Alzheimer’s Disease Neuroimaging Initiative 2, *AUC* area under the receiver operating characteristic curve

Cutoffs were chosen on the basis of those used in AD clinical trials and diagnostic studies: “Anti-Amyloid Treatment in Asymptomatic Alzheimer’s Disease” (6–18 = asymptomatic AD); “A Placebo-controlled, Double-blind, Parallel-group, Bayesian Adaptive Randomization Design and Dose-Regimen-find Study to Evaluate Safety, Tolerability and Efficacy of BAN2401 in Subjects with Early Alzheimer’s Disease” (50–64 years: ≤15; 65–69 years: ≤12; 70–74 years: ≤11; 75–79 years: ≤9; 80–90 years ≤7; and ADNI 2 (16 years of education: normal ≥9; early MCI 9–11; AD ≤8; 8–15 years of education: normal ≥5; early MCI 5–9; AD ≤4; 0–7 years of education: normal ≥3; early MCI 3–6; AD ≤2)

#### PPV and NPV: normal cognition versus MCI

Table 3 provides PPVs and NPVs for MMSE cutoff scores used in AD clinical trials and diagnostic studies. Of note, we provide a range of MMSE cutoff scores other than ≤24 and/or ≤26 to determine the accuracy of other scores and to potentially facilitate decision-making regarding optimal cutoff score use. The MMSE score of 26 yielded a PPV and NPV of 64.6 % and 68.2 %, respectively, suggesting this cutoff is associated with a >35 % chance that NACC subjects may be inaccurately classified as having MCI or AD dementia. MMSE scores <26 increased in PPV and declined in NPV.

**Table 3:** PPV and NPV for AD clinical trial and diagnostic study MMSE cutoff scores for normal cognition versus MCI and AD dementia in NACC

MMSE score	Sensitivity	Specificity	PPV	NPV
Normal cognition (*n* = 10,741) versus MCI (*n* = 5883)				
18	0.4	100.0	85.2	64.4
19	0.6	99.9	86.0	64.4
20	1.0	99.8	78.7	64.5
21	1.6	99.8	79.8	64.6
22	2.8	99.6	79.9	64.9
23	4.8	99.3	78.7	65.3
24	8.4	98.5	75.9	66.0
25	13.5	96.8	69.9	66.8
Cutoff score = 26	21.7	93.4	64.6	68.2
27	33.5	85.9	56.9	69.9
Normal cognition (*n* = 10,741) versus AD dementia (*n* = 6814)				
18	29.1	100.0	99.8	66.5
19	33.2	99.9	99.8	67.8
20	38.8	99.8	99.4	69.7
21	45.0	99.8	99.3	71.9
22	52.0	99.6	98.9	74.5
23	59.3	99.3	98.3	77.4
Cutoff score = 24	67.0	98.5	97.0	80.8
25	67.0	98.5	97.0	80.8
MCI (*n* = 5883) versus AD dementia (*n* = 6814)				
18	29.1	99.4	98.4	52.3
19	33.2	99.0	97.6	53.7
20	38.8	98.4	96.8	55.7
21	45.0	97.2	95.3	58.0
22	52.0	95.2	93.3	60.8
23	59.3	91.6	90.1	63.8
Cutoff score = 24	59.3	91.6	90.1	63.8
25	74.7	78.3	81.5	70.7

*Abbreviations*: *NACC* National Alzheimer’s Coordinating Center, *MMSE* Mini Mental State Examination, *MCI* mild cognitive impairment, *AD* Alzheimer’s disease, *PPV* positive predictive value, *NPV* negative predictive value

PPV and NPV are based on the prevalence of MCI and AD dementia in this sample. We present values beginning at cutoffs commonly used for determining MCI (≤26) and AD dementia (≤24) in clinical trials and diagnostic studies. MMSE scores <18 continued to decline in NPV and increase in PPV and thus are not presented

#### PPV and NPV: normal cognition versus AD dementia

In the comparison between normal cognition and AD dementia, there was a PPV of 97.0 % for the MMSE cutoff of ≤24, but the NPV was only 80.8 %. The NPV suggests there is a 19.2 % chance that NACC subjects with AD dementia are not detected by this cutoff.

#### PPV and NPV: MCI versus AD dementia

Relative to normal cognition versus AD dementia, the PPV and NPV for the MMSE cutoff of 24 were lower for distinguishing between MCI and AD dementia (90.1 % and 63.8 %, respectively).

#### MMSE and CDR score agreement

The level of agreement between the MMSE and CDR scores improved across the AD spectrum (*p* < 0.001 for all). Using MMSE cutoffs previously validated to discriminate across CDR scores [12], κ values were the worst for questionable dementia or MCI (κ = 0.15, slight agreement). There was fair agreement for normal cognition (κ = 0.37), as well as mild (κ = 0.27) and moderate (κ = 0.33) dementia. There was moderate agreement for severe dementia (κ = 0.48).

### AUC, PPV, and NPV for LM-II cutoff scores

#### AUC

The accuracy of all LM-II cutoff scores was clinically inadequate for distinguishing between normal cognition and MCI (AUC <0.75 for nearly all) and was worst for the BAN2401 trial cutoff of ≤15 in 50–64-year-olds (AUC 0.61). A similar pattern emerged between MCI and AD dementia. In terms of normal cognition versus AD dementia, the LM-II cutoff of ≤15 in the BAN2401 trial for 50–64-year-olds was only 0.64; the AUC for all remaining LM-II cutoffs was >0.75.

#### PPV and NPV: normal cognition versus MCI

Tables 4, 5, and 6 present PPVs and NPVs for LM-II cutoff scores for the A4 and BAN2401 trials and ADNI 2. The suggested LM-II cutoffs had a high probability of inaccurate NACC diagnosis. The age-adjusted LM-II cutoff of ≤15 used in the BAN2401 trial resulted in a 33.1 % probability that NACC subjects with MCI, aged 50–64 years, actually had MCI. NPV was lowest (72.8 %) for the LM-II cutoff used for 80–90-year-olds. There was a similar pattern for the remaining age categories. Regarding ADNI 2 cutoffs that defined MCI, the PPV reached as low as 52.2 % for NACC subjects with 16+ years of education. The NPV was <50.0 % for all LM-II cutoffs among NACC subjects with 0–7 years of education.

**Table 4:** PPV and NPV for AD clinical trial and diagnostic study LM-II cutoff scores for normal cognition versus MCI in NACC

	Sensitivity	Specificity	PPV	NPV
LM-II score (A4 Trial) (10,741 normal cognition; 5883 MCI)				
Cutoff score = 6	40.5	91.1	71.4	73.6
LM-II score (BAN2401 Trial)				
50–64 years (2315 normal cognition; 897 MCI)				
Cutoff score = 15	92.1	27.8	33.1	90.1
65–69 years (2092 normal cognition; 909 MCI)				
Cutoff score = 12	77.7	52.0	41.3	84.3
70–74 years (2083 normal cognition; 1182 MCI)				
Cutoff score = 11	78.1	57.3	50.9	82.1
75–79 years (1824 normal cognition; 1270 MCI)				
Cutoff score = 9	67.0	70.0	60.9	75.2
80–90 years (2212 normal cognition; 1472 MCI)				
Cutoff score = 7	54.8	80.7	65.4	72.8
LM-II score (ADNI 2)				
16+ years of education (6583 normal cognition; 3202 MCI)				
Cutoff score = 9	66.4	80.2	62.1	83.0
Cutoff score = 10	73.4	73.0	57.0	84.9
Cutoff score = 11	79.8	64.4	52.2	86.7
8–15 years of education (4061 normal cognition; 2576 MCI)				
Cutoff score = 5	35.6	91.3	72.2	69.1
Cutoff score = 6	44.0	86.4	67.2	70.9
Cutoff score = 7	52.5	80.4	62.9	72.7
Cutoff score = 8	60.2	73.1	58.7	74.3
Cutoff score = 9	68.4	64.5	55.0	76.3
0–7 years of education (51 normal cognition; 85 MCI)				
Cutoff score = 3	33.7	82.4	76.3	42.4
Cutoff score = 4	44.2	76.5	76	44.8
Cutoff score = 5	54.7	74.5	78.3	49.4

*Abbreviations*: *NACC* National Alzheimer’s Coordinating Center, *MMSE* Mini Mental State Examination, *LM-II* Wechsler Memory Scale Logical Memory-II, *MCI* mild cognitive impairment, *ADNI 2* Alzheimer’s Disease Neuroimaging Initiative 2, *PPV* positive predictive value, *NPV* negative predictive value

PPV and NPV are based on the prevalence of MCI in each of the age or educational categories. Cutoffs were chosen on the basis of those used in AD clinical trials and diagnostic studies: “Anti-Amyloid Treatment in Asymptomatic Alzheimer’s Disease” (6–18 = asymptomatic AD); “A Placebo-controlled, Double-blind, Parallel-group, Bayesian Adaptive Randomization Design and Dose-Regimen-find Study to Evaluate Safety, Tolerability and Efficacy of BAN2401 in Subjects with Early Alzheimer’s Disease” (50–64 years: ≤15; 65–69 years: ≤12; 70–74 years: ≤11; 75–79 years: ≤9; 80–90 years ≤7); and ADNI 2 (16 years of education: normal ≥9; early MCI 9–11; AD ≤8; 8–15 years of education: normal ≥5; early MCI 5–9; AD ≤4; 0–7 years of education: normal ≥3; early MCI 3–6; AD ≤2)

**Table 5:** PPV and NPV for AD clinical trial and diagnostic study LM-II cutoff scores for normal cognition versus AD dementia in NACC

	Sensitivity	Specificity	PPV	NPV
LM-II score (A4 Trial) (10,741 normal cognition; 6814 AD dementia)				
Cutoff score = 6	89.0	91.1	86.4	92.9
LM-II score (BAN2401 Trial)				
50–64 years (2315 normal cognition; 995 AD dementia)				
Cutoff score = 15	99.4	27.8	37.2	99.1
65–69 years (2092 normal cognition; 722 AD dementia)				
Cutoff score = 12	98.5	52.0	41.4	99.0
70–74 years (2083 normal cognition; 1082 AD dementia)				
Cutoff score = 11	97.3	57.3	54.2	97.6
75–79 years (1824 normal cognition; 1562 AD dementia)				
Cutoff score = 9	96.2	70.0	73.3	95.6
80–90 years (2212 normal cognition; 2263 AD dementia)				
Cutoff score = 7	93.9	80.7	83.3	92.9
LM-II score (ADNI 2)				
16+ years of education (6583 normal cognition; 3118 AD dementia)				
Cutoff score = 8	92.4	90.9	82.8	96.2
8–15 years of education (4061 normal cognition; 3518 AD dementia)				
Cutoff score = 4	84.4	94.6	93.1	87.5
0–7 years of education (51 normal cognition; 143 AD dementia)				
Cutoff score = 2	77.8	88.2	94.9	58.4

*Abbreviations*: *NACC* National Alzheimer’s Coordinating Center, *MMSE* Mini Mental State Examination, *MCI* mild cognitive impairment, *LM-II* Wechsler Memory Scale Logical Memory-II, *ADNI 2* Alzheimer’s Disease Neuroimaging Initiative 2, *PPV* positive predictive value, *NPV* negative predictive value

PPV and NPV are based on the prevalence of AD dementia in each of the age or educational categories. Cutoffs were chosen on the basis of those used in AD clinical trials and diagnostic studies: “Anti-Amyloid Treatment in Asymptomatic Alzheimer’s Disease” (6–18 = asymptomatic AD); “A Placebo-controlled, Double-blind, Parallel-group, Bayesian Adaptive Randomization Design and Dose-Regimen-find Study to Evaluate Safety, Tolerability and Efficacy of BAN2401 in Subjects with Early Alzheimer’s Disease” (50–64 years: ≤15; 65–69 years: ≤12; 70–74 years: ≤11; 75–79 years: ≤9; 80–90 years ≤7); and ADNI 2 (16 years of education: normal ≥9; early MCI 9–11; AD ≤8; 8–15 years of education: normal ≥5; early MCI 5–9; AD ≤4; 0–7 years of education: normal ≥3; early MCI 3–6; AD ≤2)

**Table 6:** PPV and NPV for AD clinical trial and diagnostic study LM-II cutoff scores for MCI versus AD dementia in NACC

	Sensitivity	Specificity	PPV	NPV
LM-II score (A4 Trial) (5883 MCI; 6814 AD dementia)				
Cutoff score = 6	89.0	51.8	68.1	80.3
LM-II score (BAN2401 Trial)				
50–64 years (897 MCI; 995 AD dementia)				
Cutoff score = 15	99.4	5.3	53.9	88.9
65–69 years (909 MCI; 722 AD dementia)				
Cutoff score = 12	98.5	16.2	48.2	93.1
70–74 years (1182 MCI; 1082 AD dementia)				
Cutoff score = 11	97.3	15.9	51.5	86.7
75–79 years (1270 MCI; 1562 AD dementia)				
Cutoff score = 9	96.2	26.2	61.5	85.0
80–90 years (1472 MCI; 2263 AD dementia)				
Cutoff score = 7	93.9	37.8	69.8	80.2
LM-II score (ADNI 2)				
16+ years of education (3202 MCI; 3118 AD dementia)				
Cutoff score = 8	92.4	48.3	63.5	86.8
8–15 years of education (2576 MCI; 3518 AD dementia)				
Cutoff score = 4	84.4	64.4	76.4	75.1
0–7 years of education (85 MCI; 143 AD dementia)				
Cutoff score = 2	77.8	66.3	79.6	64.0

*Abbreviations*: *NACC* National Alzheimer’s Coordinating Center, *MMSE* Mini Mental State Examination, *MCI* mild cognitive impairment, *LM-II* Wechsler Memory Scale Logical Memory-II, *ADNI 2* Alzheimer’s Disease Neuroimaging Initiative 2, *PPV* positive predictive value, *NPV* negative predictive value

PPV and NPV are based on the prevalence of AD dementia in each of the age or educational categories. Cutoffs were chosen on the basis of those used in AD clinical trials and diagnostic studies: “Anti-Amyloid Treatment in Asymptomatic Alzheimer’s Disease” (6–18 = asymptomatic AD); “A Placebo-controlled, Double-blind, Parallel-group, Bayesian Adaptive Randomization Design and Dose-Regimen-find Study to Evaluate Safety, Tolerability and Efficacy of BAN2401 in Subjects with Early Alzheimer’s Disease” (50–64 years: ≤15; 65–69 years: ≤12; 70–74 years: ≤11; 75–79 years: ≤9; 80–90 years ≤7); and ADNI 2 (16 years of education: normal ≥9; early MCI 9–11; AD ≤8; 8–15 years of education: normal ≥5; early MCI 5–9; AD ≤4; 0–7 years of education: normal ≥3; early MCI 3–6; AD ≤2)

#### PPV and NPV: normal cognition versus AD dementia

The PPV for an LM-II score of 15 in 50–64-year-old NACC subjects was 37.2 %, and it remained similar for the other age categories. In terms of ADNI 2 cutoffs for individuals with 16+ years of education, there was a 17.2 % chance that NACC subjects who did not have AD dementia scored at or below the ADNI 2 defined score for AD dementia. NPV was >90.0 % for almost all AD dementia LM-II cutoffs, although the cutoff for NACC subjects with 0–7 years of education had an NPV of 58.4 %.

#### PPV and NPV: MCI versus AD Dementia

PPV was 53.9 % for the LM-II cutoff score of 15 in the 50–64-year-old group and 48.2 % for the cutoff of 12 in the 65–69-year-old group. Almost all other PPVs were below 70.0 %. NPV reached as low as 64.0 % for the LM-II score of 2 in the 0–7 years of education group.

## Discussion

Nearly 60 % of currently active and recruiting phases II and III AD clinical trials and diagnostic studies rely on MMSE scores to determine inclusion, and several use LM-II test scores. MMSE and LM-II cutoffs used to determine eligibility were associated with a high probability of inaccurate diagnostic classification in the >23,000 NACC subjects with normal cognition, MCI, and AD dementia. In the NACC sample, the MMSE and LM-II cutoff scores lacked diagnostic accuracy for the identification of MCI, and LM-II cutoffs poorly distinguished AD dementia from MCI (AUC <0.75). It was the consistently low PPVs and NPVs for MMSE and LM-II cutoff scores across all diagnostic classifications that were most alarming, however. The PPV was only 64 % for the MMSE cutoff often used to define MCI (or early AD dementia) in AD trials and diagnostic studies. The PPVs and NPVs were remarkably low for LM-II cutoffs and spanned the entire AD spectrum, with many PPVs and NPVs below 50 %, and as low as 33 % and 42 %, respectively. Given NACC’s large sample and representativeness of the target clinical trial population (NACC is an important recruitment source for AD trials), there is a strong possibility that many of the multicenter studies in which investigators are testing AD therapeutic and diagnostic methods include subjects from a nontarget population. Such inappropriate sampling could potentially lead to biased or inaccurate results.

The psychometric limitations of the MMSE and LM (e.g., large ceiling and floor effects, learning biases) limit their diagnostic accuracy. The MMSE and LM are highly influenced by demographic factors (e.g., age, education) [11, 31–33]. Many patients with MCI and mild AD dementia can perform within the “normal” range, and cognitively intact individuals can frequently score within the impaired range (healthy older adults commonly exhibit impaired retention on the WMS) [16]. Subjects in AD trials and diagnostic studies are likely to have had repeated exposure to the MMSE and LM, given that they are among the most widely used measures in the clinical management of AD and are included in many research registries (e.g., NACC ADCs). Both instruments are sensitive to practice effects and subsequent inflated scores; in fact, past work has shown that subjects with MCI from ADNI demonstrated practice effects only for LM [34]. It is the psychometric weaknesses of the MMSE and LM-II that underpin their lack of utility as stand-alone diagnostic measures, and their isolated use to determine inclusion into AD trials and diagnostic studies could lead to inappropriate sampling and affect the validity and reliability of study results [18, 19].

There was an overall lack of agreement between MMSE and CDR scores among NACC subjects. The CDR is considered the gold standard for staging dementia severity, and the MMSE appears to lack validity in detecting and discriminating across the various disease stages, particularly at the mild end of the spectrum [35]. This has significant economic, clinical, and research implications, given that clinical trials rely on the MMSE to distinguish between subjects with normal cognition, MCI, and AD dementia when determining inclusion. Moreover, national guidelines in parts of the world use the MMSE to define dementia severity to guide pharmacological intervention [36, 37].

There is a need to improve upon current study inclusion methods for AD clinical trials and diagnostic studies. The continued use of existing brief, inexpensive methods for determining entry into AD trials may lead to inaccurate study findings, including failure to meet endpoints, because of inappropriate inclusion of subjects into the trials, rather than lack of efficacy of the compounds being studied. With the tremendous amount of time and financial resources devoted to the development of new and cutting edge AD therapeutics and diagnostic methods, it may be time to set aside this “penny-wise and pound-foolish” approach to the selection of screening and/or selection instruments and devote adequate time and resources to the development of rigorous new measures that may be expensive and time-consuming, but would increase the accurate detection of the appropriate population and do justice to the science at hand. This would require development of specific tests based on the specific goal of the screening, focusing on cognitive (or other) domains, with appropriate variability (diminished floor and ceiling effects), extensive normative and clinical data, and attention to cultural and language differences. Alternatively, there are existing instruments and methodology that could be implemented that may facilitate appropriate enrollment. For example, a recent study in ADNI found optimal diagnostic certainty of MCI included <1 standard deviation below normative reference on two episodic memory measures (LM-II and Auditory Verbal Learning Test delay recall) [38]. If AD clinical trials and diagnostic studies continue to rely on single screening measures due to their time- and cost-effectiveness, it is encouraged that more stringent criterion cutoffs be used [39] and the methodology employed in this paper be used (i.e., calculation of PPV, NPV) to identify the best cutoffs for accurate diagnostic classification using existing measures. However, instead of relying on the MMSE, more comprehensive screening tools, such as the Montreal Cognitive Assessment (MoCA), may be helpful. The MoCA has been shown to demonstrate greater predictive ability of dementia and have lower ceiling effects than the MMSE [40, 41]. In sum, the specific solution for optimizing inclusion methods for AD trials is unclear, but alternatives need to be considered, including the possibility of abandoning current practice and developing new methods.

The present study is not without limitations. The generalizability of the PPVs and NPVs is restricted to the NACC sample, a convenience research sample. However, this concern is attenuated, given that many clinical trials and diagnostic studies recruit from NACC sites. The cross-sectional data may have precluded robust diagnostic status within the NACC sample. Longitudinal studies are needed to clarify the limitations of the MMSE and LM across diagnostic severity groups, including their sensitivity to change and the role of practice effects, particularly in the context of recent work from the NACC dataset that shows most pronounced practice effects over time (relative to other measures from the UDS) on tasks of semantic and episodic memory [42].

## Conclusions

The use of MMSE and LM-II scores to determine eligibility for AD clinical trials and diagnostic studies may lead to inappropriate inclusion or exclusion of subjects. Such biases in sample selection could translate to misleading results from trials testing the efficacy of new AD treatments, or diagnostic studies examining methods and biomarkers for the detection of AD.

## Abbreviations

AD: Alzheimer’s disease
ADC: Alzheimer’s Disease Center
ADNI: Alzheimer’s Disease Neuroimaging Initiative
AUC: area under the receiver operating characteristic curve
CDR: Clinical Dementia Rating
LM: Logical Memory
MCI: mild cognitive impairment
MMSE: Mini Mental State Examination
MoCA: Montreal Cognitive Assessment
NACC: National Alzheimer’s Coordinating Center
and NPV: negative predictive value
PPV: positive predictive value
ROC: receiver operating characteristic
SD: standard deviation
UDS: Uniform Data Set
WMS: Wechsler Memory Scale
